# Predictive factors for esophageal stenosis in patients receiving prophylactic steroid therapy after endoscopic submucosal dissection for esophageal squamous cell carcinoma

**DOI:** 10.1186/s12876-024-03135-9

**Published:** 2024-01-20

**Authors:** Junichi Mizuno, Yuji Urabe, Shiro Oka, Hirona Konishi, Kazuki Ishibashi, Motomitsu Fukuhara, Hidenori Tanaka, Akiyoshi Tsuboi, Ken Yamashita, Yuichi Hiyama, Takahiro Kotachi, Hidehiko Takigawa, Ryo Yuge, Toru Hiyama, Shinji Tanaka

**Affiliations:** 1 Department of Gastroenterology, Graduate School of Biomedical & Science, Hiroshima University, Hiroshima, Japan; 2 Department of Gastrointestinal Endoscopy and Medicine, Hiroshima University Hospital, 1-2-3 Kasumi, Minamiku, Hiroshima, 734-8551 Japan; 3 Department of Endoscopy, Hiroshima University Hospital, Hiroshima, Japan; 4 Department of Hiroshima Clinical Research and Development Support Center, Hiroshima University Hospital, Hiroshima, Japan; 5 Health Service Center, Hiroshima University, Hiroshima, Japan

**Keywords:** Esophageal squamous cell carcinoma, Esophageal stenosis, Prednisolone, Steroids, Triamcinolone acetonide

## Abstract

**Background:**

Methods to prevent esophageal stenosis (ES) after endoscopic submucosal dissection (ESD) for superficial esophageal squamous cell carcinoma (ESCC) have received increasing attention. Although steroid administration is a prophylactic treatment, the risk factors for ES during prophylactic steroid therapy remain unknown. Therefore, this study aimed to retrospectively evaluate the risk factors for refractory ES in patients administered prophylactic steroids after ESD for ESCC.

**Methods:**

Among 795 patients with ESCC (854 lesions), 180 patients (211 lesions) administered local triamcinolone acetonide (TrA) and/or oral prednisolone were recruited for this study. We compared the total number of endoscopic balloon dilatation (EBD) procedures performed for post-ESD ES and clinical findings (tumor size, ESD history or chemoradiation therapy [CRT], entire circumferential resection, muscle layer damage, supplemental oral prednisolone administration, EBD with TrA injection, and additional CRT) between patients with refractory and non-refractory ES. EBD was continued until dysphagia resolved. We categorized cases requiring ≥ 8 EBD procedures as refractory postoperative stenosis and divided the lesions into two groups.

**Results:**

Multivariate logistic regression analysis revealed that factors such as ESD history, CRT history, tumor size, and entire circumferential resection were independently associated with the development of refractory ES. The withdrawal rates of EBD at 3 years were 96.1% (52/53) and 58.5% (39/59) in the non-refractory and refractory groups, respectively.

**Conclusions:**

Our data suggest that entire circumferential resection and CRT history are risk factors for refractory post-ESD ES in ESCC, even with prophylactic steroid administration.

**Supplementary Information:**

The online version contains supplementary material available at 10.1186/s12876-024-03135-9.

## Background

Esophageal carcinoma is a common cause of mortality worldwide [1], and its incidence has increased recently. The two types of esophageal cancer include esophageal squamous cell carcinoma (ESCC) and adenocarcinoma. ESCC has one of the worst prognoses among all cancer types; however, it can be radically treated when detected early. In particular, stage 0 ESCC can be treated via endoscopic resection [2]. In general, the larger the ESCC tumor size, the higher the likelihood of cancer evolving into an advanced stage. However, ESCC occasionally progresses by spreading laterally into the mucosal or submucosal layer. This superficial spreading type of ESCC rarely invades the muscular layer; thus, this lesion type can be resected endoscopically.

Endoscopic submucosal dissection (ESD) is an advanced surgical procedure that involves the use of endoscopy to resect gastrointestinal tumors before they penetrate the muscular layer. The advantage of ESD is that an en bloc resection can be theoretically achieved for a large lesion. Studies have reported the usefulness of ESD for treating superficial ESCC (SESCC) [3–7].

Esophageal stenosis (ES) often occurs after ESD for SESCC [8, 9]. According to the Japanese Guidelines for the Diagnosis and Treatment of Carcinoma of the Esophagus 2017, SESCCs covering more than two-thirds of the circumference are excluded from the category of lesions with absolute indications for ESD [10]. Recently, an expanded adaptation of ESD for SESCCs was attempted using steroids. Triamcinolone acetonide (TrA) injection administered into the ulcer base after ESD and oral steroid therapy with prednisolone may prevent ES [11]. The disadvantage of oral steroid therapy with prednisolone is the risk of systemic adverse effects, whereas TrA injection administration may cause muscle injury or perforation. Patients with severe ES risk after ESD can receive both TrA injection and oral steroid therapy with prednisolone [12]. The ES rate reportedly decreased in endoscopically resected ulcers, measuring three-quarters of the subtotal of the circumference when receiving steroid prophylaxis versus no prophylaxis [13]. However, the risk factors for ES during prophylactic steroid therapy are unknown. Patients who developed ES after these prophylactic methods were treated using endoscopic ballooning dilation (EBD). However, in some refractory cases, multiple EBD sessions may be required for ES release. Few studies have examined the risk factors for refractory ES associated with steroid prophylaxis after ESD for SESCC. Therefore, based on long-term prognosis, our study aimed to retrospectively evaluate the risk factors for refractory ES in patients who received prophylactic steroid therapy after ESD for SESCCs.

## Methods

### Aim, design, and setting

This study aimed to retrospectively evaluate the risk factors for refractory ES in patients who received prophylactic steroid therapy after ESD for SESCCs. The study was retrospective in design and conducted at Hiroshima University Hospital between January 2010 and December 2018.

### Study population

**Fig. 1:**
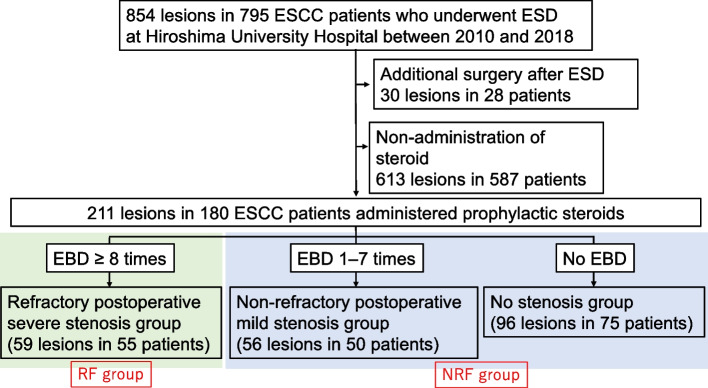
Study flowchart. The flowchart shows the outcomes of patients with no or mild stenosis (NRF group, blue box) and those with severe stenosis (RF group, green box) after prophylactic steroid therapy. The division of RF and NRF groups was determined using the median number of dilatations required in EBD cases (≥ 8 times/0–7 times). EBD was repeated until dysphagia symptoms resolved. *EBD* Endoscopic balloon dilatation, *ESCC* Esophageal squamous cell carcinoma, *ESD* Endoscopic submucosal dissection, *NRF* Non-refractory, *RF* Refractory

We retrospectively examined the data of 795 patients with 854 lesions who underwent ESD for SESCC between January 2010 and December 2018 at Hiroshima University Hospital. Of these, 587 patients with 613 lesions who did not receive steroids to prevent stenosis were excluded. Additionally, 28 patients with 30 lesions who underwent an additional operation after ESD were excluded (Fig. 1). Ultimately, 180 patients with 211 lesions were included in this study. Tumor morphology was observed under white light endoscopy and classified according to the Paris classification. Magnification endoscopy with narrow-band imaging and ultrasound endoscopy were subsequently performed to diagnose tumor depth in all cases.

### Ethical approval

Informed consent for cancer treatment was offered and accepted by all patients, and written informed consent was obtained from all patients for participation in this study. This study was approved by the Institutional Review Board of Hiroshima University Hospital (E2022-0079).

### Procedures for esophageal ESD

In all patients, esophageal ESD was performed by experienced endoscopists who had performed ESD for gastric cancer > 20 times or expert gastrointestinal endoscopists certified by the Japanese Society of Gastroenterological Endoscopy. ESD was performed using a single-channel upper gastrointestinal endoscope with a water-jet system (GIF-Q260 J; Olympus, Tokyo, Japan; or EG-450RD5 or EG-590WR; Fujifilm, Tokyo, Japan) and transparent cap (D-201–11804; Olympus, Tokyo, Japan) attached to the endoscope tip. The primary electrosurgical knives were DualKnife (Olympus, Tokyo, Japan) and Hook Knife (Olympus, Tokyo, Japan) until 2015, after which DualKnife J (Olympus, Tokyo, Japan) and SB Knife Jr. (Sumitomo Bakelite, Tokyo, Japan) were used. The VIO300D (ERBE Elektromedizin GmbH, Tubingen, Germany) electrosurgical generator was used. During the procedure, marking dots were placed outside the lesion margins with iodine staining, and then glycerin (10%) and indigo carmine (0.005%) solutions were injected. DualKnife or DualKnife J was used to make a circumferential mucosal incision on the oral side of the lesion around the periphery of the marking dots. A hook knife or SB Knife Jr was used to dissect the submucosal layer from the oral to anal side of the lesion after injecting 0.4% hyaluronic acid solution (MucoUp; Seikagaku Co., Tokyo, Japan). During ESD, carbon dioxide insufflation was used to reduce patient discomfort and prevent serious injury from perforation [14]. Intra/postoperative bleeding was controlled using hemostatic forceps (Coagrasper; Olympus, Tokyo, Japan) in the soft coagulation mode of VIO300D. Procedure-related perforation was identified endoscopically or by the presence of free air on a plain chest radiograph. Endoscopic clipping was performed to treat intraoperative perforation.

### Management of ES after ESD

In all cases expected to result in a post-ESD iatrogenic ulcer greater than half the circumference, we injected TrA into the ulcer base after ESD. Each base was injected diffusely (approximately 1 cm apart) with 0.2 mL of TrA (10 mg/mL) and approximately 10 mL within the remaining submucosal layer of the post-ESD ulcer base using a 25-gauge, 4-mm needle (TOP Corporation, Tokyo, Japan). This was performed during ESD and 2 or 3 days after ESD.

Oral prednisolone was administered with TrA in all cases where the post-ESD iatrogenic ulcer was expected to cover most or the entire circumference, except in patients with hepatitis B infection [15], severe diabetes mellitus, osteoporosis, or any immunodeficiency. Oral prednisolone administration generally began on the third postoperative day (0.5 mg/kg/day), with a gradually tapering dose (5 mg/week) as previously reported [16, 17].

The degree of swallowing was evaluated using the dysphagia score (0 = able to eat a normal diet and have no dysphagia, 1 = able to swallow some solid foods, 2 = able to swallow only semi-solid foods, 3 = able to swallow only liquid, and 4 = unable to swallow anything/complete stricture) every 4 weeks [18]. If patients experienced dysphagia in the 4 weeks between outpatient examinations, they were treated for ES removal according to the protocol.

When patients reported symptoms with a dysphagia score of > 1, EBD was performed after confirming the inability to pass a single-channel scope (GIF-Q260J, GIF-H260Z, or GIF-H 290Z; Olympus Medical Systems, Tokyo, Japan). EBD sessions were performed bi-weekly until the symptoms resolved or a single-channel scope (GIF-Q260J; Olympus Medical Systems, Tokyo, Japan) could pass through the esophagus. If patients reported symptoms with a dysphagia score of ≤ 2, EBD was performed after confirming the inability to pass a single-channel scope. EBD was performed using a through-the-scope balloon (CRE; Boston Scientific, Natick, MA, USA). In most cases, we selected a 10–12- or 12–15-mm balloon and increased the expansion pressure gradually. Since 2013, patients were diffusely injected with 4 mL of TrA 10 mg/mL (eight administrations of 0.5 mL each) into any EBD-induced laceration; before 2013, steroids were not administered.

### Assessment

Patient (age, sex, a history of esophageal ESD, and chemoradiation therapy [CRT] history), tumor (size, location, gross type, and depth), and treatment (extent of resection, muscle layer damage, route of steroid administration, and administration of additional CRT after ESD) factors were assessed retrospectively. The mean number of EBD procedures needed for ES removal was 14.5 ± 18.5 (0–104, median 8) (Table 1). In this study, we defined refractory as a median of ≥ 8 dilated cases. We then divided the 211 lesions into 2 groups: 59 lesions in 55 patients with refractory postoperative stenosis who had undergone EBD ≥ 8 times (RF group) and 152 lesions in 125 patients with non-refractory postoperative stenosis who had undergone 0–7 EBD procedures (NRF group) (Fig. 1). The NRF group included 56 lesions in 50 patients with mild postoperative stenosis who had undergone 1–7 EBD procedures and 96 lesions in 75 patients with no postoperative stenosis (Fig. 1). Muscle layer damage was defined as a perforation or scratched appearance of the muscularis propria. The entire circumferential diameter (longitudinal diameter) was measured from the oral to anal edges of the resected specimens. The withdrawal rates of EBD were examined, with 56 lesions in 50 patients in the NRF group and 59 lesions in 55 patients in the RF group. In this study, we followed up patients until 2021.

**Table 1:** Patient characteristics

Characteristics	*N* = 211
Male sex, n (%)	189 (89.6)
Age (years), mean ± SD	68.3 ± 9.5
ESD history, n (%)	42 (19.9)
CRT history, n (%)	17 (10.5)
Tumor size, mean ± SD	37.7 ± 19.3
Location, n (%)	
Ce/Ut	47 (22.2)
Mt	101 (47.9)
Lt/Ae	63 (29.9)
Macroscopic type, n (%)	
0-Is	7 (3.3)
0-IIa	11 (5.2)
0-IIb	16 (7.6)
0-IIc	177 (83.9)
Tumor depth (clinical diagnosis), n (%)	
cT1a-EP/LPM	159 (75.4)
cT1a-MM/ cT1b-SM1	43 (20.4)
cT1b-SM2	9 (4.2)
Tumor depth (pathological diagnosis), n (%)	
pT1a-EP	56 (26.5)
pT1a-LPM	85 (40.3)
pT1a-MM	46 (40.3)
pT1b-SM1	12 (5.6)
pT1b-SM2	12 (5.6)
Presence of additional CRT after ESD, n (%)	27 (12.8)
Total number of EBD procedures, median (range)	8 (0–104)
Entire circumferential ESD, n (%)	47 (22.3)
Muscle layer injury, n (%)	16 (7.5)
Prevention methods for ES	
TrA injection only	89 (42.2)
TrA injection + oral PSL	122 (57.8)
EBD with TrA injection, n (%)	52^a^ (46.4)

^a^This percentage indicates the proportion of EBD with TrA injection cases to the total number of EBD cases

### Statistical analyses

Categorical variables, presented as numbers (%), were statistically compared using the chi-square and Fisher’s exact tests. Continuous variables, presented as means ± standard deviations or as medians (ranges), were compared using Student’s t-test. We analyzed all variables in multivariate logistic regression analysis. The Kaplan–Meier method was performed to calculate the EBD withdrawal rate. Statistical analyses were performed using JMP version 15 (SAS Institute Inc, Cary, North Carolina, USA). *P*-values of < 0.05 were considered statistically significant.

## Results

Table 1 summarizes the clinicopathological characteristics of the 211 lesions in 180 patients. The rate of R0 resection was 83.4% (176/211); however, no residual recurrent lesions were observed. No side effects, such as infections, hyperglycemia, iatrogenic Cushing syndrome, or bleeding during the oral steroid period, were observed in this study.

Ce/Ut, Cervical esophagus/upper thoracic esophagus; CRT, Chemoradiation therapy; EBD, Endoscopic balloon dilatation; ESD, Endoscopic submucosal dissection; EP/LPM, Epithelial/lamina propria; Lt/Ae, Lower thoracic esophagus/abdominal esophagus; MM/SM1, Muscularis mucosae; Mt, Middle thoracic esophagus; SD, Standard deviation; SM2/SM3, Submucosa 2/submucosa 3; TrA, Triamcinolone acetonide.

Seventy-five patients (96 lesions) did not develop ES (Fig. 1), whereas 105 patients (115 lesions) developed ES. Table 2 presents the results of univariate analysis comparing clinicopathological factors (ESD history, CRT history, tumor location, macroscopic type, tumor size, resection area, muscle layer damage, prophylactic methods of stenosis, pathological tumor depth, and additional CRT after ESD) between the two groups.

**Table 2:** Comparison of clinicopathological factors between the NRF and RF groups

Variable	NRF group	RF group	*p*-value
	(*n* = 152)	(*n* = 59)	
ESD history			0.101
+	26 (17.1)	16 (27.1)	
−	126 (82.9)	43 (72.9)	
CRT history			0.0233
+	8 (5.3)	9 (15.3)	
−	144 (94.7)	50 (84.7)	
Location			0.753
Ce-Ut	33 (21.7)	14 (23.7)	
Mt-Ae	119 (78.3)	45 (76.3)	
Macroscopic type			0.601
0-Is/0-Iia	12 (7.9)	6 (10.2)	
0-IIb/0-Iic	140 (92.1)	53 (89.8)	
Tumor size (mm), mean ± SD	34.4 ± 17.8	46.2 ± 20.3	< 0.01
Resection area			< 0.01
Entire circumference	19 (12.5)	28 (47.5)	
Sub-circumference	133 (87.5)	31 (52.5)	
Muscle layer damage			0.375
+	13 (8.6)	3 (5.1)	
−	139 (91.4)	56 (94.9)	
Prophylactic methods of stenosis			0.0649
TrA injection only	70 (46.0)	19 (32.2)	
TrA injection + oral PSL	82 (54.0)	40 (67.8)	
Pathological tumor depth			0.729
pT1a	134 (88.2)	53 (89.8)	
pT1b	18 (11.8)	6 (10.2)	
Additional CRT after ESD			0.225
+	22 (14.5)	5 (8.5)	
−	130 (85.5)	54 (91.5)	

*EBD* Endoscopic balloon dilatation, *ESD* Endoscopic submucosal dissection, *CRT* Chemoradiation therapy, *NRF* Non-refractory, *PSL* Prednisolone, *RF* Refractory, *SD* Standard deviation, *TrA* Triamcinolone acetonide

Longitudinal tumor size (NRF group vs. RF group: 34.4 ± 17.8 vs. 46.2 ± 20.3, *p *< 0.01), CRT history (8/144 vs. 9/50, *p* = 0.023), and entire circumferential resection (19/133 vs. 28/31, *p* < 0.01) differed significantly between the two groups; however, no significant differences were observed in other factors between the two groups. After multiple logistic regression analysis, ESD history, CRT history, longitudinal tumor size, and entire circumferential resection were independently associated with the development of refractory ES (Table 3).

**Table 3:** Multivariate analysis to determine factors associated with refractory esophageal stenosis

Clinicopathological factor	OR	95% CI	*p*-value
Presence of ESD history	3.77	1.56–9.08	0.0031
Presence of CRT history	4.19	1.26–13.96	0.0196
Ce-Ut of location	1.19	0.43–3.28	0.874
0-Is/0-IIa	1.39	0.40–4.80	0.598
Tumor size (≤ 40, > 40 mm)	2.71	1.18–6.19	0.0180
Presence of muscle injury	0.52	0.11–2.45	0.415
Entire circumferential resection	5.51	2.38–12.78	< 0.0001
pT1b	0.82	0.21–3.15	0.778
Presence of additional CRT after ESD	0.57	0.16–2.00	0.383
TrA injection + oral PSL use	1.80	0.84–3.84	0.126

*CI* Confidence interval, *CRT* Chemoradiation therapy, *EBD* Endoscopic balloon dilatation, *OR* Odds ratio, *PSL* Prednisolone, *TrA* Triamcinolone acetonide

Four cases of entire circumference resection without ES were observed. Two of these cases were whole circumferential lesions and two of sub-whole circumferential lesions. The two sub-whole circumferential lesions had a portion of the whole circumference resection < 1 cm in diameter. We examined clinicopathological factors of refractory stenosis for each prophylactic method, as different treatment strategies for steroid administration have different effects on stenosis (Additional files 1 and 2). In patients in whom ES was prevented via TrA injection only, CRT history (NRF group vs. RF group: 3/67 vs. 4/5, *p* = 0.03) and longitudinal tumor size (NRF group vs. RF group: 33.4 ± 17.6 vs. 45.0 ± 16.9, *p *< 0.0159) differed significantly between the two groups. However, in patients who prevented ES via TrA injection and oral prednisolone intake, the longitudinal tumor size (NRF vs. RF group: 35.2 ± 18.2 vs. 46.7 ± 22.1, *p* < 0.01) and resection area (10/72 vs. 22/18, *p* < 0.01) differed significantly between the two groups. Moreover, the effect of TrA injection during EBD on refractory stenosis was examined between the group that underwent ≥ 8 EBDs and the group that underwent 1–7 EBDs, but no significant association was found between refractory stenosis and EBD with TrA injection (EBD 1–7 times, EBD ≥ 8 times: 27/56 vs. 24/35, *p* = 0.416).

**Fig. 2:**
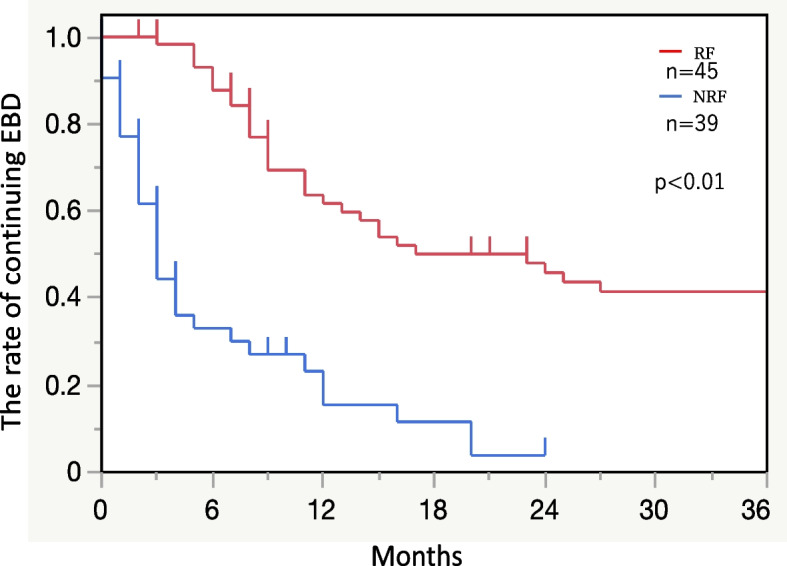
Kaplan–Meier curve for the withdrawal rate of EBD after ESD. The withdrawal rates of EBD in the NRF and RF groups 1 year after ESD were 86.5% (77/114) and 38.3% (26/59), respectively, and 3 years after ESD were 96.1% (52/53) and 58.5% (39/59), respectively. EBD, Endoscopic balloon dilatation; ESD, Endoscopic submucosal dissection; NRF, Non-refractory; RF, Refractory

The withdrawal rate of EBD in the NRF group was significantly higher than that in the RF group (*p* < 0.01). The withdrawal rate of EBD in the NRF and RF groups was 86.5% (77/114) and 38.3% (26/59), respectively, 1 year after ESD and was 96.1% (52/53) and 58.5% (39/59), respectively, 3 years after ESD (Fig. 2).

## Discussion

Our study showed that ESD history, CRT history, tumor size, and entire circumferential resection were clinicopathological findings associated with an increased risk of refractory ES in patients who underwent prophylactic steroid therapy after ESD for SESCC. We also determined that the withdrawal rate of EBD over 3 years after ESD was 96.1% in the NRF group and 58.5% in the RF group.

Studies have reported the risk factors for ES after ESD. Chen et al. identified endoscopic mucosal resection/ESD history, circumferential diameter resection, non-en bloc resection, submucosal infiltration, and circumferential resection range as independent risk factors for post-ESD ES [19]. A meta-analysis reported the following lesion characteristics as risk factors for ES after ESD: involvement of the upper third of the esophagus, macroscopic type 0–IIa/IIc, tumor invasive depth of pT1a-MM/pT1b-SM, longitudinal length > 5 cm, entire circumference lesion, and circumferential range > 3/4 [20]. In addition, we previously identified muscle layer damage and longitudinal mucosal defect length ≥ 5 cm as risk factors for refractory postoperative stenosis after entire circumferential esophageal ESD [16]. This study identified entire circumferential resection as an independent risk factor and longitudinal tumor size as a risk factor for post-ESD refractory ES after receiving steroids. According to the Japanese esophageal endoscopic mucosal resection/ESD guidelines, lesions that both measured > 5 cm longitudinally and presented as entire circumferential epithelium/lamina propria ESCC were not indicated for ESD, and our results support these guidelines [21].

The effectiveness of TrA injection on an EBD-induced laceration in ES after ESD is unknown; however, TrA injection is reportedly useful in postoperative ES [22]. Hanaoka et al. [22] reported that adding a steroid injection into the EBD-induced laceration significantly decreased the number of EBDs required to resolve the strictures. Moreover, Xiang et al. [23] reported the usefulness of administering a steroid injection with radial incision and cutting for refractory ES after esophageal ESD. However, our data did not support the usefulness of TrA injection into the EBD-induced laceration for preventing refractory postoperative stenosis. This result suggests that ES from ESD is larger than that from esophagectomy. A randomized controlled trial is required to elucidate the effects of TrA injection on the EBD-induced lacerations.

CRT is one of the most effective treatments for ESCC, and treatment of cT1N0M0 ESCC with CRT has response rates > 90% [24]. In addition, prophylactic CRT for patients with “pMM, vascular invasion, and negative margins” or “pSM and negative margins” after endoscopic resection showed an overall survival rate of 90.7% (90% confidence interval: 84.0–94.7) after 3 years [25]. Moreover, in that study, esophageal strictures of grade ≥ 3 (CTCAE v4.0) were found in only 0.6% of patients. We previously reported that only 34% of grade 2 and 6% of grade 3 ES occurred after salvage radiotherapy for SESCC following non-curative endoscopic resection [26]. Thus, prophylactic CRT after ESD may not be a risk factor for refractory esophageal stricture in patients at low risk of stricture. However, this study identified patients with a history of CRT for esophageal cancer as being at a high risk of refractory esophageal stricture. A previous long-term study of definitive concurrent CRT for patients with pT1N0M0 found that grades 2–4 ES occurred in 11% of patients and metachronous ESCC in 22% of patients [27]. As CRT irradiates the esophagus, mild fibrosis and loss of peristaltic function of the esophagus may occur in the submucosa. Therefore, the healing process for ESD-induced mucosal defects after CRT is considered aberrant and thus prone to ES.

Subgroup analyses using prophylactic methods of stenosis revealed that CRT history was associated with refractory ES in patients who prevented ES via TrA injection only, whereas entire circumferential resection was associated with refractory ES in patients who prevented ES via TrA injection and oral prednisolone intake. These results suggest that CRT history is associated with refractory ES in cases where the endoscopist would not expect stenosis to occur and that entire circumferential stenosis developed even with strong stenosis prevention.

Clinically, restenosis often appears after releasing ESD-induced stenosis and is occasionally found after several months. As a result of a > 1-year follow-up of refractory ES cases after esophageal ESD, administering prophylactic steroids is reportedly ineffective in reducing the number of EBD procedures and improving long-term outcomes [28]. Therefore, long-term observation is important during ES release. However, few studies have examined long-term observations after the EBD release of ES. In this study, most patients in the NRF group and approximately 60% of patients with ESCC in the RF group who received prophylactic steroid therapy after ESD and were treated with continuous EBD showed successful resolution of ES. Moreover, the withdrawal rate of EBD after ESD in the RF group increased by approximately 20% from year 1 to 3. This result shows the importance of consistent EBD in refractory ES cases.

This study has some limitations. This was a single-center, retrospective study. A large-scale, multicenter prospective study should be conducted to confirm our results. Additionally, the interval between EBDs was not fixed; therefore, patients with persistent or recurrent stenosis are more likely to have more frequent EBDs and be assigned to the refractory group. Moreover, because patients were enrolled over a long period, the endoscopist’s skills and techniques may have improved over time, which may have influenced treatment outcomes. Although a principal protocol for the endoscopic dilatation procedure exists, the principal protocol is not always consistent and can change depending on the circumstances. In this study, no precise criteria were established for the indication of prophylactic steroid administration, which was at the discretion of each physician. Therefore, preoperative predictions could have been made regarding risk factors for refractory stenosis. A prospective randomized controlled trial is needed to validate the results of this study.

## Conclusions

ES should be carefully prevented, especially in ESD cases of patients with SESCC and CRT history or entire circumferential resection. These two characteristics were identified as risk factors for refractory ES after ESD. Therefore, such patients should not only receive oral prednisolone and TrA injection, but a prolonged course of oral prednisolone should also be considered. Moreover, new preventive ES techniques, such as the TrA-filling method [29] and covered esophageal stents [30], should be considered for these at-risk patients.

### Supplementary Information

**Additional file 1:** **Supplementary Table 1.** Comparison of clinicopathological factors between the groups in patients administered TrA injection only.

**Additional file 2: Supplementary Table S2.** Comparison of clinicopathological factors between the groups in patients administered TrA injection and oral prednisolone.

## Data Availability

All data generated or analyzed during this study are included in this article. Further inquiries can be directed to the corresponding author.

## Abbreviations

CRT: Chemoradiation therapy
EBD: Endoscopic balloon dilatation
ES: Esophageal stenosis
ESCC: Esophageal squamous cell carcinoma
ESD: Endoscopic submucosal dissection
NRF: Non-refractory stenosis
RFI: Refractory stenosis
SESCC: Superficial esophageal squamous cell carcinoma
TrA: Triamcinolone acetonide
