# Supplementary material for: Predictive factors for esophageal stenosis in patients receiving prophylactic steroid therapy after endoscopic submucosal dissection for esophageal squamous cell carcinoma
Source: BMC Gastroenterol. 2024 Jan 20;24:41. doi: 10.1186/s12876-024-03135-9 (PMC10799525; doi:10.1186/s12876-024-03135-9)
Supplement: Supplementary file 1 — Additional file 1: Supplementary Table 1. Comparison of clinicopathological factors between the groups in patients administered TrA injection only. [file 12876_2024_3135_MOESM1_ESM.docx]

| **Supplementary Table 1.** Comparison of clinicopathological factors between the groups in patients administered TrA injection only | | | |
| --- | --- | --- | --- |
| Variable | NRF group | RF group | p-value |
|  | (n=70) | (n=19) |  |
| ESD history |  |  | 0.0535 |
| + | 11 (15.7) | 7 (36.8) |  |
| − | 59 (84.3) | 12 (63.2) |  |
| CRT history |  |  | 0.03 |
| + | 3 (4.3) | 4 (21.1) |  |
| − | 67 (95.7) | 15 (78.9) |  |
| Location |  |  | 0.054 |
| Ce-Ut | 20 (28.6) | 10 (52.6) |  |
| Mt-Ae | 50 (71.4) | 9 (47.4) |  |
| Macroscopic type |  |  | 0.219 |
| 0-Is/0-IIa | 7 (10.0) | 4 (21.1) |  |
| 0-IIb/0-IIc | 63 (90.0) | 15 (78.9) |  |
| Tumor size (mm), mean±SD | 33.4±17.6 | 45.0±16.9 | 0.0159 |
| Resection area |  |  | 0.0683 |
| Entire circumference | 9 (12.9) | 6 (31.6) |  |
| Sub-circumference | 61 (87.1) | 13 (68.4) |  |
| Muscle layer damage |  |  | 0.858 |
| + | 3 (4.3) | 1 (5.3) |  |
| - | 67 (95.7) | 18 (94.7) |  |
| Pathological tumor depth |  |  | 0.795 |
| pT1a | 64 (91.4) | 17 (89.5) |  |
| pT1b | 6 (8.6) | 2 (10.5) |  |
| Additional CRT after ESD |  |  | 0.316 |
| + | 9 (12.9) | 1 (5.3) |  |
| - | 61 (87.1) | 18 (94.7) |  |
| CRT, Chemoradiation therapy; EBD, Endoscopic balloon dilatation; ES, Esophageal stenosis; ESD, Endoscopic submucosal dissection; NRF, Non-refractory; RF, Refractory; SD, Standard deviation; TrA, Triamcinolone acetonide | | | |
