# Supplementary material for: Predictive factors for esophageal stenosis in patients receiving prophylactic steroid therapy after endoscopic submucosal dissection for esophageal squamous cell carcinoma
Source: BMC Gastroenterol. 2024 Jan 20;24:41. doi: 10.1186/s12876-024-03135-9 (PMC10799525; doi:10.1186/s12876-024-03135-9)
Supplement: Supplementary file 2 — Additional file 2: Supplementary Table S2. Comparison of clinicopathological factors between the groups in patients administered TrA injection and oral prednisolone. [file 12876_2024_3135_MOESM2_ESM.docx]

| **Supplementary Table S2.** Comparison of clinicopathological factors between the groups in patients administered TrA injection and oral prednisolone | | | |
| --- | --- | --- | --- |
| Variable | NRF group | RF group | p-value |
|  | (n=82) | (n=40) |  |
| ESD history |  |  | 0.586 |
| + | 15 (18.3) | 9 (22.5) |  |
| − | 67 (81.7) | 31 (77.5) |  |
| CRT history |  |  | 0.24 |
| + | 5 (6.1) | 5 (12.5) |  |
| − | 77 (93.9) | 35 (87.5) |  |
| Location |  |  | 0.369 |
| Ce-Ut | 13 (15.9) | 4 (10.0) |  |
| Mt-Ae | 69 (84.1) | 36 (90.0) |  |
| Macroscopic type |  |  | 0.805 |
| 0-Is/0-IIa | 5 (6.1) | 2 (5.0) |  |
| 0-IIb/0-IIc | 77 (93.9) | 38 (95.0) |  |
| Tumor size (mm), mean±SD | 35.2±18.2 | 46.7±22.1 | <0.01 |
| Resection area |  |  | <0.01 |
| Entire circumference | 10 (12.2) | 22 (55.0) |  |
| Sub-circumference | 72 (87.8) | 18 (45.0) |  |
| Muscle layer damage |  |  | 0.186 |
| + | 10 (12.2) | 2 (5.0) |  |
| - | 72 (87.8) | 38 (95.0) |  |
| Pathological tumor depth |  |  | 0.467 |
| pT1a | 70 (85.4) | 36 (90.0) |  |
| pT1b | 12 (14.6) | 4 (10.0) |  |
| Additional CRT after ESD |  |  | 0.369 |
| + | 13 (15.9) | 4 (10.0) |  |
| - | 69 (84.1) | 36 (90.0) |  |
| CRT, Chemoradiation therapy; EBD, Endoscopic balloon dilatation; ES, Esophageal stenosis; ESD, Endoscopic submucosal dissection; NRF, Non-refractory; RF, Refractory; SD, Standard deviation; TrA, Triamcinolone acetonide | | | |
|  | | | |
|  | | | |
